# Polymorphisms of Leptin (-2548 G/A) and Leptin Receptor (Q223R) Genes in Iranian Women with Breast Cancer

**DOI:** 10.1155/2015/132720

**Published:** 2015-06-14

**Authors:** Reza Mahmoudi, Bahareh Noori Alavicheh, Mohammad Amin Nazer Mozaffari, Mohammad Fararouei, Mohsen Nikseresht

**Affiliations:** ^1^Cellular and Molecular Research Center, Yasuj University of Medical Sciences, Yasuj, Iran; ^2^Students Research Committee, Yasuj University of Medical Sciences, Yasuj, Iran; ^3^HIV/AIDS Research Center, Shiraz University of Medical Sciences, Shiraz, Iran

## Abstract

Recent studies have shown that polymorphisms in leptin and leptin receptor genes are associated with increased risk for breast cancer. This study aimed at investigating -2548 G/A polymorphism in leptin gene and Q223R polymorphism in leptin receptor gene in patients with breast cancer. The study included 45 women with breast cancer and 41 healthy women. PCR-RFLP was used to determine the genotype of the subjects in terms of -2548 G/A polymorphism in leptin gene and Q223R polymorphism in leptin receptor gene. Serum levels of leptin were also measured by ELISA. For -2548 G/A polymorphism, the genotypes were homozygous AA (OR = 1.13; *p* = 0.8) and heterozygous GA (OR = 0.41; *p* = 0.2) and for Q223R polymorphism, the genotypes were homozygous RR (OR = 6.7; *p* = 0.09) and heterozygous QR (OR = 8.3; *p* = 0.06). The mean serum level of leptin was 33.22 ± 21.35 ng/mL in patients and 29.49 ± 23.27 ng/mL in the normal participants (*p* = 0.3). Although, despite the magnitude of the associations, the results suggested no statistically significant contribution of -2548 G/A polymorphism (in leptin gene), Q223R polymorphism (in leptin receptor gene), and serum leptin levels in predicting the risk of breast cancer, further studies with larger sample size are suggested.

## 1. Introduction

Breast cancer is the most common type of malignancy and the second leading cause of cancer death in women, after lung cancer [1, 2]. The cause of breast cancer is still unknown; however, epidemiological evidence considers three causes including endocrine, genetic, and environmental factors. A direct relationship has been observed between obesity and increased risk of breast cancer [3]. Adipokines including adiponectin and leptin which are secreted by adipose tissue have a role in cancer biology in obese people [4]. Leptin (from Greek leptos, meaning “thin”) was discovered in 1994 following isolation of obesity gene [5]. This gene is located on chromosome 7 (7q31) [6]. Leptin (LEP) is a 16 kD glycoprotein hormone mainly secreted by adipose tissue. This hormone binds to its receptor (LEPR) in hypothalamus and plays key roles in regulating metabolism [7]. Leptin has three main effects: it increases calorie expenditure, decreases ATP production, and reduces appetite [8]. In the body, leptin acts through two types of receptors: a type of long receptors existing in some parts of the brain and hypothalamus which belongs to type I cytokine family and inhibits neuropeptide Y in the hypothalamus after activation by leptin, resulting in suppressed appetite and increased metabolism through impact on thyroid and adrenal hormones [9, 10]. The second form of leptin receptor exists in peripheral tissues such as muscle, fat, liver, and intestines and exerts many metabolic effects in tissues upon activation, regarding the energy balance and body weight [9, 11]. Leptin can bind to six receptors (leptin receptors a to f). These six isoforms of leptin receptor (obR) belong to type I cytokine receptors family [12] which are converted to six different types as obR_a–f_ through mRNA alternative splicing during gene expression [13, 14]. They consist of a long isoform (obRb), four short isoforms, and one secretory isoform [15]. Leptin receptor is produced by a gene on human chromosome 1 [16] and mouse chromosome 4 [17], and CD_295_ has been assigned to it in differentiation systems [18]. Long chain leptin receptor type b is responsible for most of the known effects of leptin which performs signaling in four different pathways: JAK-STAT (Janus kinase-signal transducer and activator of transcription), MAPK (mitogen-activated protein kinase), PI3K (phosphatidylinositol 3-kinase), and AMPK (adenosine monophosphate activated kinase) [19].

Leptin is essential for the growth of human mammary gland and recent studies have shown that it may be effective in breast cancer [20]. Leptin gene is expressed in normal breast tissue, breast cancer cell lines, and solid tumors. It is overexpressed in more than 92% of breast cancers, while this was not reported in any of the normal breast epithelial cells. In addition to stimulating cell division, leptin can transform breast cancer cells into malignant forms [21]. The expression of leptin and its receptor increases significantly in cancerous tissues [20]. Leptin plays a role in cell growth, differentiation, and angiogenesis and can increase endothelial cells products such as NO and overexpression of VEGF, FGF2, and VEGFR/2 [22]. Leptin acts also as a mitogenic and migration factor in some cells such as normal epithelial cells and malignant cells [23]. Leptin can stimulate the growth of cancer cells through expression of aromatase and production and activation of estrogen in breast cancer epithelium, resulting in reduction or inhibition of inhibitory effects of antiestrogens in proliferation of breast cancer cells [24]. Studies have shown that expression of leptin and its receptor is significantly increased in primary breast cancer with metastases [25].

Various polymorphisms occur in leptin and its receptor gene including -2548 G/A and Q223R. In -2548 G/A polymorphism, an adenine replaces guanine in nucleotides -2548 upstream from the ATG start site at 5′ region of leptin gene promoter. This polymorphism affects the expression of leptin and its secretion from adipose tissue [6]. Q223R polymorphism is the result of guanine to adenine substitution in nucleotide 668 of exon 6 from the start codon, resulting in replacement of positively charged arginine instead of neutral glutamine at amino acid 223. This polymorphism occurs in a region which encodes the extracellular domain of leptin receptor, affects the function of the receptor, and impairs the ability of leptin to bind to its receptor. This change in amino acid affects all isoforms of leptin receptor [26]. Study of these polymorphisms in different populations may show the relationship between the frequency of these polymorphisms and the risk for cancer. In fact, different populations have been studied in this regard and different results have been obtained. The present study investigated these polymorphisms in patients with breast cancer in Yasuj, Iran.

## 2. Materials and Methods

Forty-five women with breast cancer and 41 healthy women were enrolled in this study. The patients included those women who were diagnosed according to pathology and the signs of the disease, while the controls were those with negative pathology results for breast cancer. All subjects were informed about the use of the results and voluntary participation in the study, and written consents were obtained from the patients and controls. 5 mL blood sample was collected for performing PCR-RFLP and measuring leptin. Clinical information about patients, such as cancer stage, estrogen and progesterone receptors status, HER2 status, and age, was recorded.

DNA was extracted using the QIAamp DNA Mini Kit (QIAgen Inc., Santa Clarita, CA). Briefly according to the procedure of this kit, 200 *μ*L of whole human blood treated with EDTA was used for extracting of genomic DNA. All centrifugation steps are carried out at room temperature (15–25°C). 20 *μ*L QIAGEN Protease (or proteinase K) was transferred into the bottom of a 1.5 mL microtube and 200 *μ*L whole blood was added to the tube and then 200 *μ*L lysis buffer (AL buffer of the kit) was added to the sample and mixed by pulse-vortexing for 15 s and incubated at 56°C for 10 min. Then 200 *μ*L ethanol (96–100%) was added to the sample and mixed again by pulse-vortexing for 15 s. Carefully the mixture was applied to the QIAamp Mini spin column (in a 2 mL collection tube) and centrifuged at 6000 ×g (8000 rpm) for 1 min. The QIAamp Mini spin column was placed in a clean 2 mL collection tube and the tube containing the filtrate was discarded. Carefully the QIAamp Mini spin column was opened and 500 *μ*L Buffer AW1 (included in the kit) was added and then centrifuged at 6000 ×g (8000 rpm) for 1 min. Again the QIAamp Mini spin column was placed in a clean 2 mL collection tube and the collection tube containing the filtrate was discarded, and 500 *μ*L Buffer AW2 (included in the kit) was added and centrifuged at full speed (20,000 ×g; 14,000 rpm) for 3 min. The QIAamp Mini spin column was placed in a clean 1.5 mL microtube and the collection tube containing the filtrate was discarded and 200 *μ*L Buffer AE (included in the kit) added and then incubated at room temperature for 1 min and centrifuged at 6000 ×g (8000 rpm) for 1 min. A second elution step with a further 200 *μ*L Buffer AE increased yields by up to 15%. The average yield of DNA was 5.5 *μ*g and A260/A280 ratio was 1.68–1.92.

PCR-RFLP was performed for determining the genotype. Leptin concentration was measured by Mediagnost ELISA kit (Germany).

The primers indicated in Table 1 were used to amplify the regions for -2548 G/A and Q223R polymorphisms in leptin and leptin receptor genes, respectively. PCR conditions for a 25 *μ*L reaction volume were as follows: 2.5 *μ*L PCR buffer, 1 *μ*L MgCl_2_, 0.5 *μ*L dNTP mix, 1 *μ*L of each primer, 3 *μ*L template DNA, and 0.3 *μ*L Taq DNA Polymerase (Go TaqDNA Polymerase, Invitrogen).

PCR program for both polymorphisms was as follows: initial denaturation at 94°C for 4 minutes; 40 cycles including denaturation at 94°C for 30 s, annealing at 58°C for 30 s, and extension at 72°C for 30 s; and final extension at 72°C for 10 min. To confirm amplification of the desired fragments, all samples were electrophoresed on 1.5% agarose gel after PCR. The samples were then digested with* MspI* enzyme (New England Biolabs) for leptin receptor gene and* Hha1* enzyme for leptin gene for 16 h. After the incubation period, the products were electrophoresed again on 2% agarose. Regarding Q223R LEPR polymorphism, the presence of only one band of 416 bp length indicated individuals with QQ homozygous genotype, the presence of three bands of 416 bp, 291 bp, and 125 bp length indicated individuals with QR heterozygous genotype, and the presence of two bands of 125 bp and 291 bp length indicated individuals with RR homozygous genotype.

Regarding -2548 G/A LEP polymorphism, the presence of only one band of 241 bp length indicated individuals with GG homozygous genotype, the presence of three bands of 241 bp, 181 bp, and 60 bp length indicated individuals with GA heterozygous genotype, and the presence of two bands of 181 bp and 60 bp length indicated individuals with AA homozygous genotype.

*t*-test was used to compare the means and ANOVA and logistic regression were used to study the relationship between different genotype of -2548 G/A LEP polymorphism and Q223R LEPR polymorphism with breast cancer and leptin level. In all calculations, the probability level of *p* < 0.05 was deemed statistically significant. Statistical analyses were performed using SPSS-16.

## 3. Results

This study consisted of 45 women with breast cancer (mean age = 47.09 ± 11.45 years) and 41 normal subjects (mean age = 48.37 ± 8.80 years).

### 3.1. Relationship between Serum Leptin Levels and Breast Cancer

The mean level of serum leptin was 33.22 ± 21.35 ng/mL in the patient group and 29.49 ± 23.27 ng/mL in the normal group. Comparison of serum leptin levels in patients and controls revealed no significant difference in the serum level of this hormone between the healthy subjects and the patients (*p* = 0.3).

### 3.2. Relationship between -2548 G/A LEP Polymorphism and Q223R LEPR Polymorphism with the Risk of Breast Cancer and Serum Leptin

Distributions of the genotypes and frequencies of the alleles of -2548 G/A LEP polymorphism and Q223R LEPR polymorphism in breast cancer patients and healthy controls are represented in Table 2.

According to Table 2 for leptin gene, the prevalence of allele A in the patient and control groups was 72.2% and 64.6%, respectively (OR = 1.35; *p* = 0.33). No significant association was found between the risk of breast cancer and homozygous GG, homozygous AA (OR = 1.13; *p* = 0.8), and heterozygous GA (OR = 0.41; *p* = 0.2).

Regarding genotype distribution of Q223R LEPR polymorphism in normal subjects, 6 were homozygous RR (14.6%), 18 heterozygous QR (43.9%), and 17 homozygous QQ (41.5%). The genotype distribution of Q223R LEPR polymorphism in patients was 1 homozygous RR (2.2%), 25 heterozygous QR (55.6%), and 19 homozygous QQ (42.2%). According to Table 2 for leptin receptor gene, the frequency of allele R in the patient and control groups was 30% and 36.6%, respectively (OR = 0.71; *p* = 0.33). No significant association was found between homozygous QQ with homozygous RR (OR = 6.7; *p* = 0.09) and with heterozygous QR (OR = 8.3; *p* = 0.06) and risk of breast cancer.

The relationship between different genotypes of -2548 G/A LEP and Q223R LEPR polymorphisms with plasma leptin levels was investigated (Table 3) and no significant association was found between the levels of the hormone, -2548 G/A LEP polymorphism, and Q223R LEPR polymorphism and breast cancer (*p* > 0.05).

In addition, the frequency of -2548 G/A LEP and Q223R LEPR polymorphisms and pathologic parameters in breast cancer patients (Table 4) was not significantly associated (*p* > 0.05).

## 4. Discussion and Conclusion

The present study examined the relationship between -2548 G/A polymorphism in leptin gene and Q223R polymorphism in leptin receptor gene with the risk for breast cancer in Yasuj, Iran.

Breast cancer is the most common type of malignancy and the second leading cause of cancer death in women, so that its mortality rate was estimated approximately 25% until April 2013 [27]. Adipokines including adiponectin and leptin which are secreted by adipose tissue have a role in cancer biology in obese people [4]. Mutations in leptin and leptin receptor can decrease the leptin receptor signals [28]. According to the literature, mutations in this gene are associated with cancer in humans. It has been shown recently that leptin and its receptor are involved in the processes leading to the initiation and progression of breast cancer [29]. Several polymorphisms in leptin gene and its receptor gene have been related to breast cancer, such as -2548 G/A and Q223R, because -2548 G/A polymorphism in the promoter of leptin gene can affect the expression of this gene and Q223R polymorphism in leptin receptor gene is associated with impaired binding of leptin to its receptor, affecting its whole activity [30, 31].

The effect of these polymorphisms on the risk of breast cancer has been studied in different populations. Woo et al. (2006) found no significant correlation between four polymorphisms in leptin receptor gene and the risk of breast cancer. They also observed no significant difference in serum leptin levels between patients and controls. The results of this study showed that the difference of mean serum leptin levels between various genotype groups of Q223R polymorphism was not significant. In the present study also no significant correlation was found between Q223R polymorphism and breast cancer risk; in addition, no significant difference was observed in the mean serum leptin levels between patients and controls and the mean difference in serum leptin levels was not significant between different genotype groups of Q223R polymorphism [32]. Consistent with results of the present study, no relationship was observed between serum leptin concentrations and the risk of breast cancer in studies by Mantzoros et al. (1999) and Petridou et al. (2000) [33, 34]. He et al. (2012), in a comprehensive study performed as a meta-analysis, and Liu et al. (2011) found no significant association between -2548 G/A LEP polymorphism and Q223R LEPR polymorphism with the risk of breast cancer in most ethnic communities; this finding is consistent with the results obtained in the present study [35, 36]. By contrary, Snoussi et al. (2006) showed a significant association between Q223R polymorphism and -2548 G/A polymorphism with the risk of breast cancer [6]. They also reported that allele A was more frequent in patients with larger tumor size, while in the present study, no significant correlation was observed between pathological indicators and allele frequency in -2548 G/A LEP polymorphism and Q223R LEPR polymorphism [6]. In other studies, Han et al. in China (2008) and Anuradha et al. in India (2012) showed that Q223R LEPR polymorphism is associated with the risk of breast cancer; this finding is in contradiction with the results obtained in the present study [26, 37]. In 2013, He et al. studied Q223R LEPR and -2548 G/A LEP polymorphisms in patients with different cancers. Their results showed that individuals with AA genotype in leptin gene are likely to develop various cancers, especially prostate cancer. In addition, no relationship was found between Q223R LEPR polymorphism and the risk of different types of cancer. Also in this study, no significant differences were found between the expressions of mRNA of leptin and its receptor in different genotypes and different ethnicities [38].

The results of our study showed that -2548 G/A LEP and Q223R LEPR polymorphisms were highly polymorphic in the study population. The frequency of allele R in Q223R LEPR polymorphism was 0.366 in our study population which was higher than the figures reported in Pima Indian (0.32) and Arab (0.34) populations and lower than the figures reported in Caucasian (0.45) and Asian (0.85) populations [39].

However, Chung et al. reported a little difference in R allele frequency of Q223R LEPR polymorphism according to racial groups [40]. It is believed that small sample size and ethnic composition in USA have affected this result. The frequency of the allele A in -2548 G/A LEP polymorphism was 0.646 in our study population. In a study by Fan and Say, allele A was reported more frequent in Asian populations, at least among Indian, Chinese, and Malays/Peninsular Bumiputras [41].

The differences in the findings of similar studies and the present study can be attributed to various genetic polymorphisms in similar metabolic pathways (gene-gene interaction). Other factors such as environmental factors (e.g., stress and changes in environmental temperature) can affect leptin expression as confounders [42] and alter the risk of breast cancer. Also, this difference in various studies can be attributed to racial differences; a factor in a region and in a specific race may be a predisposing factor for developing certain diseases, and it may be not decisive in the second race in a different geographical area. In other words, genetic correlation is population dependent and shows different results in different populations.

In this study, no significant association was found between -2548 G/A LEP polymorphism, Q223R LEPR polymorphism, and serum leptin concentrations with the risk of breast cancer in the study population. Further studies are required to accept or reject the conclusion and to achieve more comprehensive results. In this regard, similar studies with larger sample size are recommended.

## Figures and Tables

**Table 1:** Characteristics and sequences of primers for leptin and leptin receptor genes.

Primer name	Primer sequence
LEPRF	5′ACCCTTTAAGCTGGGTGTCCCAAATAG3′
LEPRR	5′AGCTAGCAAATATTTTGTAAGCAATT3′
LEPF	5′TTTCCTGTAATTTTCCCGTGAG3′
LEPR	5′AAAGCAAAGACAGGCATAAAAA3′

**Table 2:** Final results of -2548 G/A LEP and Q223R LEPR polymorphisms in the two groups of patients and controls with the risk of breast cancer.

Genotypes	Controls *n* = 41 (%)	Patients *n* = 45 (%)	Odds ratio	95% confidence interval	*p* value
*LEP* (-2548) G/A					
GG	5 (12.2)	7 (15.6)^*∗*^	1		
GA	19 (46.3)	11 (24.4)	0.41	(0.1–1.62)	0.2
AA	17 (41.5)	27 (60)	1.13	(0.3–4.15)	0.8

Alleles					
G-allele	29 (35.4)	25 (27.8)			
A-allele	53 (64.6)	65 (72.2)	1.35	(0.74–2.44)	0.33

LEPR Q223R					
QQ	17 (41.5)	19 (42.2)	1^*∗*^		
QR	18 (43.9)	25 (55.6)	8.3	(0.92–75.36)	0.06
RR	6 (14.6)	1 (2.2)	6.7	(0.73–61.5)	0.09

Alleles					
Q-allele	52 (63.4)	63 (70)			
R-allele	30 (36.3)	27 (30)	0.71	(0.36–1.41)	0.33

^*∗*^Reference group.

**Table 3:** Comparison of the mean leptin levels in different genotypes of -2548 G/A LEP polymorphism and Q223R LEPR polymorphism in the study population.

Genotypes	Number	Mean leptin concentration ± SD (ng/mL)	*p* value
*LEP* (-2548) G/A			
AA	44	32.14 ± 20.65	0.78
GA	30	30.98 ± 23.86	
GG	12	30.04 ± 25.41	

LEPR Q223R			
RR	7	32.86 ± 22.84	0.54
QR	43	34.34 ± 23.90	
QQ	36	27.70 ± 20	

**Table 4:** Relationship between -2548 G/A LEP and Q223R LEPR polymorphisms with pathological indicators in patients with breast cancer.

Pathological feature	Number of patients (%)		*p* value
*LEP* (-2548) G/A	GA + AA	GG	

ER			
Negative	12 (92.3)^*∗*^	1 (7.7)	0.65
Positive	26 (81.3)	6 (18.8)	
PR			
Negative	13 (86.7)	2 (13.3)	1.00
Positive	25 (83.3)	5 (16.7)	
HER2			
Negative	27 (90)	3 (10)	0.19
Positive	11 (73.3)	4 (26.7)	
Stage			
I & II	21 (91.3)	2 (8.7)	0.24
III & IV	17 (72.28)	5 (22.73)	

LEPR Q223R	QR + RR	QQ	

ER			
Negative	4 (30.8)	9 (69.2)	0.5
Positive	15 (46.9)	17 (56.7)	
PR			
Negative	6 (40)	9 (60)	1.00
Positive	25 (83.3)	17 (56.7)	
HER2			
Negative	14 (46.7)	16 (53.3)	0.5
Positive	5 (33.3)	10 (66.7)	
Stage			
I & II	10 (43.4)	13 (56.5)	1.00
III & IV	9 (41)	13 (59)	

^*∗*^Numbers in parentheses represent the percentage.
